# The ATA’s Principles for Delivering Telerehabilitation Services

**DOI:** 10.63144/ijt.2026.6767

**Published:** 2026-06-01

**Authors:** American Telemedicine Association

**Affiliations:** Washington DC, USA

**Keywords:** American Telemedicine Association, Habilitation, Rehabilitation, Telehealth, Telepractice

## Abstract

Telehealth is a broad term used to describe the use of electronic or digital information and communications technologies (ICT) to deliver clinical healthcare, patient and professional health-related education, and public health and health administration. More recently, the term digital health has emerged to refer to the use of digital technologies as tools and systems that contribute to improving healthcare outcomes (i.e., artificial intelligence and machine learning, digital therapeutics, mHealth, remote patient monitoring, remote therapeutic monitoring, wearable devices, and other health analytics and information software systems). These technologies enhance the efficiency of health care delivery through the broad use of information and communication technologies (ICT) and in some commercial applications include telehealth. This document is a revision of the American Telemedicine Association’s (ATA) “Principles for delivering Telerehabilitation Services” (2017) and reflects the current utilization of telerehabilitation services. Its purpose is to inform and assist practitioners in providing effective and secure telerehabilitation services that are based on client needs, current empirical evidence, and available technologies. Rehabilitation professionals, in conjunction with professional associations and other organizations are encouraged to use this document as a resource for developing discipline-specific standards, guidelines, and practice requirements. This guide is not intended to replace the primary practitioner’s clinical decision-making about the appropriate course of healthcare for any client.

## The ATA’s Principles for Delivering Telerehabilitation Services

**This document is a revision of the American Telemedicine Association’s (ATA) Principles for Delivering Telerehabilitation Services (2017) and reflects the current utilization of telerehabilitation services.** Its purpose is to inform and assist practitioners in providing effective and secure telerehabilitation services that are based on client needs, current empirical evidence, and available technologies. Rehabilitation professionals, in conjunction with professional associations and other organizations, are encouraged to use this document as a resource for developing discipline-specific standards, guidelines, and practice requirements. **This guide is not intended to replace the primary practitioner’s clinical decision-making about the appropriate course of healthcare for any client.**

**The material in this guide should not be interpreted nor used as a legal standard of care and should be viewed in the context of evolving global health policies and regulations concerning telerehabilitation. The content of this document, while divided into administrative, clinical, technical, and ethical content areas, ideally should be considered in its entirety.**

## Scope and Definitions

Telehealth is a broad term used to describe the use of electronic or digital information and communications technologies (ICT) to deliver clinical healthcare, patient and professional health-related education, and public health and health administration. Telehealth services can be synchronous/real-time interactive, or asynchronous/store-and-forward or hybrid (i.e., a combination of the two), combining both approaches to offer flexibility in care delivery.

More recently, the term digital health has emerged to refer to the use of digital technologies as tools and systems that contribute to improving healthcare outcomes (i.e., artificial intelligence and machine learning, digital therapeutics, mHealth, remote patient monitoring, remote therapeutic monitoring, wearable devices, and other health analytics and information software systems) by enhancing the efficiency of healthcare delivery through the broad use of information and communication technologies and in some commercial publications including telehealth. The terms “telehealth” and “digital health” should not be thought of as interchangeable in meaning when addressing state and federal policies, standards of practice, or regulations. They represent distinct concepts and both enable health and healthcare systems to become more efficient, patient-centered, and data-driven. Telehealth regulatory policies include digital technologies.

Terminology used to describe telerehabilitation is similarly broad. Some terms specifically refer to individual rehabilitation disciplines, (e.g., teleaudiology, telespeech, tele-occupational therapy, and tele-physical therapy). More generic terms, such as teletherapy, telehealth (endorsed by the American Occupational Therapy Association and the American Physical Therapy Association), and telepractice (endorsed by the American Speech-Language-Hearing Association) are also used, allowing for a broader focus on populations and activities, such as educational settings and wellness promotion in addition to rehabilitation. It is not the intent of this document to resolve the debate over terminology; rather to provide consistency across applications, regardless of vocabulary. For the purposes of this document, the term ‘telerehabilitation’ will be used. The reader is reminded that terminology may differ according to the application and across practice settings and business models.

Telerehabilitation refers to the delivery of rehabilitation and habilitation services via a variety of information and communication technologies (ICT) or commonly referred to as, “telehealth” technologies. Clinically, the term “telerehabilitation” encompasses a range of rehabilitation and habilitation service types that include evaluation, assessment, monitoring, prevention, intervention, supervision, education, consultation, and coaching. Telerehabilitation services can be deployed across all patient populations and multiple healthcare settings including clinics, homes, schools, or community-based worksites. ICT used to deliver and manage rehabilitation and habilitation services may incorporate—but are not limited to— artificial intelligence (AI) and machine learning systems, augmented reality, chat messaging, mobile health applications, patient portals or platforms, remote therapeutic monitoring software, robotics, therapeutic gaming technologies, video and audio conferencing, virtual reality, wearable sensor technologies, and other health data analytics such as electronic health records (EHRs). ICT usage in the delivery of rehabilitation services is expected to change as technology continues to evolve.

Telerehabilitation services are delivered to adults and children by a broad range of professionals. These professionals may include, but are not limited to, audiologists, nurses, occupational therapists, physical therapists, physicians, psychologists, rehabilitation engineers, speech-language pathologists, and educators. Other personnel such as paraprofessionals, family members, caregivers and telehealth navigators may play a key role in facilitating telerehabilitation encounters. These individuals may assist as telepresenters, helping with technical setup, patient communication, supporting real time interaction, and ensuring smooth operational workflow.

Telerehabilitation contributes to achieving the goals of value-based care by improving patient outcomes, enhancing care coordination, increasing access to timely care, and reducing avoidable healthcare utilization and overall healthcare costs by rewarding professionals for creating efficiencies in healthcare delivery.

For the purposes of this document, the term ‘professionals’ will be used to refer specifically to rehabilitation professionals delivering telerehabilitation services, while acknowledging the essential contributions of supporting personnel who help optimize the telehealth experience. The term “clients” will be used to refer to all recipients of telerehabilitation services and is intended to include both children and adults.

This document contains requirements, recommendations, or actions that are identified by text containing the keywords “shall,” “may,” and “shall not.” “Shall” indicates a required action whenever feasible and practical under local conditions. These indications are found in bold throughout the document. “May” indicates additional points that may be considered to further optimize the healthcare process. “Shall not” indicates that this action is strongly advised against.

## Key Principles

The following information represents key administrative, clinical, technical, and ethical principles that should be considered in the deployment or integration of telerehabilitation services. Some principal statements may be repeated in more than one category as relevant.

As education and advocacy are central to the continued growth of telehealth, this document serves as a resource to educate members of health professions, students, stakeholders, administrators, legislators, and community members. Rehabilitation professionals, in conjunction with professional associations and other organizations, are encouraged to use this document for guidance in developing discipline-specific standards, guidelines, professional development or training, and practice requirements. Professional specific telehealth, telepractice or telerehabilitation-related guidance documents can be found on the websites of the American Occupational Therapy Association, American Physical Therapy Association, and the American Speech-Language-Hearing Association. Telerehabilitation stakeholders should also refer to the **ATA’s Resource Center** for information that pertains to additional telehealth operations.

## Administrative Principles

- Organizations and professionals **shall** be aware of and comply with laws, regulations, guidelines, and standards set forth by nationally recognized professional associations and other credentialing, privileging, accrediting, and regulatory requirements for licensing, certification, professional liability, and ongoing professional development or training for the delivery of telerehabilitation services.
- Organizations and/or professionals **shall** be aware of and comply with any federal or state laws or licensure regulations that contain language that may restrict types of services that can be delivered by telerehabilitation or promulgate any other specific limitations, requirements or standards for telerehabilitation service provision.
- Organizations and professionals **shall** be aware of all applicable models of licensure portability (e.g., Interstate Licensure Compacts, Expedited License, Limited License, etc.) that may impact interstate practice using telerehabilitation.
- Organizations and professionals **shall** be aware of and comply with any additional or necessary operational or contractual requirements or arrangements with the implementation of telerehabilitation services. Examples include contractual agreements (e.g., service level agreements, Business Associate Agreements) and credentialing requirements at the site where the practitioner is located and the site where the client is located (which may vary between states or jurisdictions).
- Organizations and/or professionals **shall** be aware of and comply with or follow any federal and state telehealth laws, professional regulations, or organizational policies that apply to informed consent. The informed consent process informs the client about the proposed scope of the telerehabilitation services during a virtual encounter and can include types of services; types of technologies; capturing of video, audio, and/or photos; use of support personnel; record keeping; privacy and security; terminating services; billing arrangements; and other parameters related to encounters.
- Organizations and professionals **shall** be aware of current billing and coding processes (e.g., the use of any designated modifiers or specific codes) that may be required by federal or state insurance plans, commercial insurers, or private health plan payment policies. Professionals **shall** be aware of various reimbursement arrangements, agreements and alternative payment methodologies that exist such as fee-for-service, bundled payment systems, primary care medical home, accountable care organizations, shared savings, partial risk and other payment models with cost savings and cost avoidance metrics. This could affect the scope of services implemented and/or the billing and coding methodologies. Furthermore, professionals **shall** be aware of evolving value-based care arrangements and models where professionals are reimbursed based on the quality of care they deliver rather than the volume of services.
- Organizations and/or professionals **shall** be aware of and comply with the advanced requirements for privacy, security, and confidentiality (e.g., HIPAA, HITECH, FERPA, etc.) associated with delivering telerehabilitation services. This **may** include orientation to an organization’s policies governing the use of telerehabilitation, the appropriate use of devices, and privacy and security considerations prior to engaging in telerehabilitation services.
- Organizations and professionals **shall** be aware of and comply with all federal, state, professional association, and healthcare entity requirements for clinical and non-clinical documentation.
- Organizations and professionals performing telerehabilitation services **shall** be aware of and inform the client that any time an automated conversation system (AI, Avatar, Voice Response, etc.) is utilized, the patient will be notified that the patient is not corresponding with a human.
- Organizations and professionals **shall** have a mechanism in place to ensure that clients are aware of their rights and responsibilities with respect to accessing telerehabilitation services and/or their personal health records including the process for communicating complaints and grievances.
- Organizations and professionals **shall** ensure that an appropriate facilitator, navigator, telepresenter, translator or e-helper (i.e., caregiver, family member, provider or another authorized individual) is available when necessary to meet client needs before, during, and after the telerehabilitation encounter. However, any additional personnel or persons accompanying or participating in the virtual encounter **shall** be announced, recognized, and approved by the client and the provider.
- Organizations and professionals **shall** establish mechanisms or policies and procedures to determine the client’s location at the time of the virtual encounter, to implement secondary modes of communication (e.g., telephone or text) in the event of technical or communication disruption during the encounter, and to promote an emergency plan to ensure the client’s safety in the event of a health-related complication, environmental emergency or hazard, or sudden natural disaster.
- Organizations and professionals engaged in telerehabilitation research **shall** ensure the protection of participants in research protocols. Research protocols **shall** be approved by the research/ethical review committee (e.g., Institutional Review Board) of the affiliated agency and be in compliance with relevant legislation, regulations, and other requirements for supporting participant decision-making, informed consent, and ethical guidelines.
- Organizations and/or professionals **shall** have in place a systematic quality improvement and performance management process that complies with any organizational, regulatory, or accrediting requirements for outcomes management. The organization **should** review the telerehabilitation program on a periodic basis to identify risks, quality of service, safety, effectiveness, and continued viability of the program. Assessment metrics **may** include quality of equipment and connectivity, client and professional satisfaction with virtual encounters, clinical outcomes, appropriateness of the virtual encounter, review of clinical documentation quality (e.g., chart review/audit), and other performance and quality metrics.
- Organizations and professionals that engage in collaborative affiliations, partnerships and/or vendor agreements **shall** be aware of applicable legal and regulatory requirements for appropriate written agreements or contracts such as, but not limited to, Business Associate Agreement (BAA), Master Service Agreements (MSA), Memorandum of Understanding (MOU), Non-Disclosure Agreement (NDA), and Statement of Work (SOW). The agreements **shall** be based on the scope and application of the telerehabilitation services offered and **shall** address the administrative, clinical, technical, ethical, and privacy requirements outlined in this document, as relevant, for all parties named.

## Clinical Principles

- Professionals **shall** be aware of and comply with laws and regulations and shall integrate guidelines and standards set forth by nationally recognized professional associations (e.g., American Speech-Language-Hearing Association, American Physical Therapy Association, and American Occupational Therapy Association) and other credentialing, privileging, accrediting, and regulatory requirements for licensing, certification, professional liability, and ongoing professional development or training for delivering telerehabilitation services.
- Professionals **shall** be aware of and comply with all professional state board telehealth or telerehabilitation regulations and any guiding scope of practice policies including any requirement for a specific verbal or written informed consent, need for in-person first visit, and/or ethical guidelines.
- Professionals who use telerehabilitation hardware, software, or devices to deliver information or services **shall** be trained in equipment and software operations and troubleshooting or readily have available the technology vendor or IT support to assist with technical difficulties. Professionals **shall** also know how to operate videoconferencing peripherals, connected devices, data sharing or accessory tools to incorporate and modify education, intervention, materials, or equipment used during an encounter.
- Professionals performing telerehabilitation services **shall** be aware of and inform the client that any time an automated conversation system (AI, Avatar, Voice Response, etc.) is utilized, the patient will be notified that the patient is not corresponding with a human.
- Professionals performing telerehabilitation services—whether synchronous (real-time interactive) or asynchronous (store-and-forward) or remote monitoring software—**shall** consider the need to modify education or intervention materials, techniques, equipment, and/or the physical setting. Regardless of any modifications made, professionals **shall** deliver services in accordance with professional standards of care, copyright laws, and the principles of evidence-based practice.
- Professionals **shall** be aware of and comply with all federal, state, industry-specific, and professional regulations and any additional healthcare entity requirements for clinical and non-clinical documentation, collection, retention, disposal, and storage of health data, health and/or medical record storage and management, and retrieval or sharing of client data or medical records to protect the client’s personal health information in accordance with federal and state regulations (e.g., HIPAA, HITECH, FERPA).
- Professionals **shall** ensure that an appropriate facilitator, telepresenter, translator, or e-helper (i.e., caregiver, family member, provider or another authorized individual) is available when necessary to meet client and provider needs before, during, and after the telerehabilitation encounter. However, any additional personnel or persons accompanying or participating in the virtual encounter **shall** be announced, recognized, and approved by the client and the provider.
- Professionals assume responsibility for ensuring the client’s safety during telerehabilitation service encounters. If during the virtual encounter, the professional observes the client might be experiencing any medical symptoms, complications, or an emergency, the virtual encounter **shall** be terminated and the client referred to an appropriate local healthcare provider or emergency service according to established policies and procedures.
- Professionals **shall** be aware of and comply with any applicable organizational or administrative telehealth guidelines impacting the delivery of telerehabilitation services.

## Technical Principles

- Organizations and professionals **shall** comply with all relevant laws, regulations, and codes for technology and technical safety.
- Organizations and professionals **shall** ensure that equipment is safe and sufficient to support diagnostic and/or treatment goals and is functioning properly at the time of clinical encounters. This includes available types of technologies or peripheral devices (e.g., additional communication devices or monitoring devices, measurement tools, other technology-based interventions, robotic devices, sound meters, wearable sensor technologies, etc.) that **may** be necessary to provide evaluations and interventions.
- Organizations and professionals **shall** have infection control policies and procedures in place for the use of telehealth equipment and peripherals that comply with organizational, and/or national, state, and local regulatory requirements. In particular, mechanisms **shall** be in place for the cleaning/sterilization of equipment for re-use by multiple clients, if applicable.
- Organizations and professionals **shall** comply with federal and state regulations (e.g., HIPAA, HITECH, FERPA) for protection of client health information and to ensure the usage of privacy and security measures for protection of data and record storage, retrieval, and transmission, and to ensure physical security of ICT hardware, software, and devices. Methods for protection of privacy and security of health information **may** include the use of authentication and/or encryption technology, audit trails, and role-based access control.
- Organizations and professionals **shall** have policies and strategies in place to address any modifications to the physical environment, hardware, software applications, and/or peripheral devices and should consider usability and accessibility factors of the client (e.g., fine/gross motor skill, behavioral aspects, cognition, speech, language, vision, and/or hearing), particularly for individuals with disabilities, to promote inclusivity and improve quality healthcare outcomes.

## Ethical Principles

- Organizations and professionals **shall** be aware of and comply with any applicable telehealth laws, regulations, statutes, and/or telerehabilitation-related policies and adhere to professional standards of practice and code of ethics.
- Organizations and professionals **shall** incorporate organizational values and client-centered ethics into policy and procedures related to telerehabilitation including education, training, and resources to address competency, access, privacy, and safety.
- Organizations and/or professionals **shall** inform clients of their rights and responsibilities when receiving rehabilitation and habilitation services through telerehabilitation, including their right to refuse or discontinue services, the benefits, risks, effectiveness, and potential outcomes of any intervention.
- Organizations and/or professionals **shall** inform clients that any time an automated conversation system (AI, Avatar, Voice Response, etc.) is utilized, the patient will be notified that the patient is not corresponding with a human.
- Organizations and/or professionals **shall** collaborate with appropriate stakeholders and other interprofessional health care providers or teams to promote quality care and safety for recipients of telerehabilitation.
- Organizations and professionals **shall** have in place a formal process for resolving ethical issues as well as policies that identify, eliminate, and reduce conflict of interest associated with the provision of telerehabilitation services.

